# 1,25-Dihydroxyvitamin D_3_ Promotes High Glucose-Induced M1 Macrophage Switching to M2 via the VDR-PPAR**γ** Signaling Pathway

**DOI:** 10.1155/2015/157834

**Published:** 2015-04-19

**Authors:** Xiaoliang Zhang, Min Zhou, Yinfeng Guo, Zhixia Song, Bicheng Liu

**Affiliations:** Institute of Nephrology, Zhong Da Hospital, Southeast University School of Medicine, Nanjing, Jiangsu 210009, China

## Abstract

Macrophages, especially their activation state, are closely related to the progression of diabetic nephropathy. Classically activated macrophages (M1) are proinflammatory effectors, while alternatively activated macrophages (M2) exhibit anti-inflammatory properties. 1,25-Dihydroxyvitamin D_3_ has renoprotective roles that extend beyond the regulation of mineral metabolism, and PPAR*γ*, a nuclear receptor, is essential for macrophage polarization. The present study investigates the effect of 1,25-dihydroxyvitamin D_3_ on macrophage activation state and its underlying mechanism in RAW264.7 cells. We find that, under high glucose conditions, RAW264.7 macrophages tend to switch to the M1 phenotype, expressing higher iNOS and proinflammatory cytokines, including TNF*α* and IL-12. While 1,25-dihydroxyvitamin D_3_ significantly inhibited M1 activation, it enhanced M2 macrophage activation; namely, it upregulated the expression of MR, Arg-1, and the anti-inflammatory cytokine IL-10 but downregulated the M1 markers. However, the above effects of 1,25-dihydroxyvitamin D_3_ were abolished when the expression of VDR and PPAR*γ* was inhibited by VDR siRNA and a PPAR*γ* antagonist. In addition, PPAR*γ* was also decreased upon treatment with VDR siRNA. The above results demonstrate that active vitamin D promoted M1 phenotype switching to M2 via the VDR-PPAR*γ* pathway.

## 1. Introduction

Chronic kidney disease (CKD), especially diabetic nephropathy (DN), is an emerging health problem that poses a growing socioeconomic burden for societies around the world [1–5]. A common pathologic feature of DN is the presence of inflammatory cells, mostly mononuclear cell infiltration occurring at early stages in the injured kidneys, followed by tubulointerstitial fibrosis at the later stages of disease progression [2–4]. Therefore, alleviating the inflammatory reaction might be a promising strategy to delay the early development of DN.

Macrophages are pivotal mediators of glomerular and tubulointerstitial inflammation and fibrosis due to their production of proinflammatory and profibrotic cytokines [2, 6, 7]. In the past years, the severity of renal inflammation and injury was thought to be correlated with the number of infiltrating macrophages [8]. However, macrophages are a heterogeneous population of cells that may undergo classical M1 activation or alternative M2 activation in response to various signals [9]. The M1 phenotype is considered to aggravate inflammation and tissue injury, and M2 macrophages play a role in the inhibition of inflammation and promotion of tissue repair [10]. Presently, mounting results tend to indicate that it is the activation state of recruited macrophages, rather than their infiltrating numbers, that finally determines the evolvement and prognosis of renal injury [11, 12]. Therefore, finding appropriate strategies to modulate macrophage phenotype and function is pivotal to the early prevention of renal injury in DN.

1,25-Dihydroxyvitamin D_3_ (vitamin D) has long been characterized as a regulator of bone and mineral homeostasis [13]. However, recent findings also demonstrated a renoprotective role of this steroid hormone [14]. Our prior research also indicated that calcitriol, a bioactive 1,25-dihydroxyvitamin D_3_, effectively decreased the enlargement of the glomerular surface area and the expansion of the glomerular mesangial matrix, alleviated podocyte effacement and proteinuria, and exerted a renoprotective role in STZ-induced diabetic nephropathy rats [15]. This protective effect extended beyond its classical regulation of mineral metabolism but was related to the regulation of macrophage phenotype. In DN rats, vitamin D not only inhibited M1 macrophage activation and abated inflammation and renal injury in the early phase but also enhanced M2 activation in the later stages to protect against renal injury [16]. However, the exact mechanism of how vitamin D switches macrophage M1-M2 phenotype is still unclear.

The pleiotropic biological activities of vitamin D are mediated by the vitamin D receptor (VDR), which is also expressed on macrophages [17–19]. However, whether vitamin D regulates macrophage phenotype by acting on VDR is not known. Recent studies also suggested that macrophage-specific peroxisome proliferator-activated receptor *γ* (PPAR*γ*) was an indispensable factor for M2 macrophage maturation, as it could control macrophage alternative activation [20, 21]. Additionally, PPAR*γ* is a primary target of vitamin D [22–26]. Therefore, in this study, we determined whether vitamin D can switch the macrophage M1 phenotype to M2 via the VDR-PPAR*γ* pathway in murine macrophage cell lines.

## 2. Materials and Methods

### 2.1. Cell Culture and Preparation

Murine macrophage cells (RAW264.7), obtained from Shanghai Bogoo Biotechnology Company (Shanghai, China), were routinely cultured in RPMI 1640 media (containing 11.1 mM glucose) supplemented with 10% fetal bovine serum (Sciencell, USA) and incubated at 37°C in 5% CO_2_. RAW264.7 cells were first stimulated with glucose in a dose- (11.1 mM, 20 mM, 25 mM, and 30 mM) and time- (0 h, 6 h, 12 h, 24 h, 36 h, and 48 h) dependent manner. The activity of intracellular iNOS was measured in order to ascertain the optimum dose and time point. A set concentration of glucose (11.1 mM) in RPMI 1640 media (Gibco, USA) was used as a control. Second, to examine the effect of vitamin D on macrophage polarization, RAW264.7 cells were incubated with 25 mM glucose for 24 h in the presence or absence of 1,25-dihydroxyvitamin D_3_ (Sigma, USA). At the same time, the classical activation models of M1 and M2 macrophages in vitro were established by treating cells with 100 U/mL IFN*γ* + 5 ng/mL LPS (M1 differentiation) (Sigma, USA) or 10 ng/mL IL-4 (M2 differentiation) (Perotech), respectively. Third, in order to explore the underlying mechanism, these cells were treated with VDR siRNA (Invitrogen, USA) and the PPAR*γ* antagonist GW9662 (Sigma, USA). The supernatants were collected, and cells were washed three times with PBS and then harvested for quantitative real-time polymerase chain reaction (RT-PCR) and western blot.

### 2.2. Real-Time PCR

Total cellular RNA was extracted from RAW264.7 cells using Trizol (TaKaRa, Japan) according to the manufacturer's instructions. All of the PCR primers were synthesized by Shanghai Generay Biotechnology Company (Shanghai, China). The primer sequences were as follows: mouse iNOS (sense: TCTTGGAGCGAGTTGTGGATGT, antisense: TAGGTGAGGGCTTGGCTGAGTG), mouse MR (sense: CCTCAGCAAGCGATGTGCCTAC, antisense: GTCCCCACCCTCCTTCCTACAA), mouse Arg-1 (sense: GGCAACCTGTGTCCTTTCTCCT, antisense: CCCAGCTTGTCTACTTCAGTCATG), mouse VDR (sense: CTTCCTAAGAGACTTCCCGAGAGA antisense: GGCATTTATTTACAGCGGTACTTGT), mouse PPAR*γ* (sense: CCACAGTTGATTTCTCCAGCATTTC, antisense: ATGCAGGTTCTACTTTGATCGCACT), and *β*-actin (sense: TGAGAGGGAAATCGTGCGTGAC, antisense: GCTCGTTGCCAATAGTGATGACC). Real-time PCR was performed on an ABI PRISM 7300 real-time PCR System (Applied Biosystems, USA). The protocol included melting for 15 minutes at 37°C and 5 seconds at 95°C and 40 cycles of two-step PCR including melting for 5 seconds at 95°C and annealing for 31 seconds at 60°C. The 2^−ΔΔ^Ct method was used to determine the relative amounts of product using *β*-actin as an endogenous control.

### 2.3. Cytokine Assays

The inducible nitric oxide synthase (iNOS) activity was assayed using an iNOS assay kit (Jiancheng, Nanjing, China). The TNF-*α*, IL-12, and IL-10 levels in supernatants were detected using Murine ELISA kits (Neobioscience, China) according to the manufacturer's instructions.

### 2.4. Treatment of Cells with siRNA

Three specific VDR siRNAs aimed at VDR mRNA were synthetized. The sequence of siRNAs is as follows: VDR siRNA-1 (sense: 5′-CCCUUCAAUGGAGAUUGCCGCAUCA-3′, antisense: UGAUGCGGCAAUCUCCAUUGAAGGG-3′), VDR siRNA-2 (sense: 5′-CCCACCUGGCUGAUCUUGUCAGUUA-3′, antisense: 5′-UAACUGACAAGAUCAGCCAGGUGGG-3′), and VDR siRNA-3 (sense: 5′-GGACAUGAUGGAACCGGCCAGCUUU-3′, antisense: 5′-AAAGCUGGCCGGUUCCAUCAUGUCC-3′). RAW264.7 macrophages were transfected with either nonspecific siRNA oligomers or stealth siRNAs targeting VDR mRNA by using the RNAiMAX reagent according to the manufacturer's instructions. The cells were seeded in 6-well dishes at 1 × 10^5^ cells/well and incubated in RPMI 1640 containing 10% FBS 24 h before transfection. When the cells were 50%~70% confluent, the old medium was removed, and the cells were washed twice with PBS before adding fresh medium. siRNA-lipid complexes containing control or VDR siRNA were formed by incubating 50 pmol of each siRNA duplex with 7.5 *μ*L of RNAiMAX for 20 min at room temperature in a total volume of 250 *μ*L of RPMI without antibiotics. The liposomes were added to the cells, and siRNA treatment was continued for 24 h. Silencing of VDR at the gene and protein level was verified by RT-PCR and western blotting.

### 2.5. Immunofluorescence Staining

For immunofluorescence, RAW264.7 cells were seeded on cover slips and allowed to adhere overnight. After incubation with different intervention reagents for 24 h, the cells were washed three times with PBS and then fixed with 4% paraformaldehyde, permeabilized in 0.5% Triton-X100 for 30 min, and blocked with 1% BSA for 1 h. Cells were washed and incubated with anti-mouse iNOS (Abcam, ab15323), MR (Abcam, ab64693), VDR (Bioss, bs-2987R), and PPAR*γ* (Bioss, bs-0530R) rabbit polyclonal antibodies overnight at 4°C. Then, cells were washed and incubated with anti-rabbit secondary antibody (Jackson, USA) for 2 h at room temperature. After staining nuclei with DAPI, cells were visualized using a IX70 fluorescence microscope (OLYMPUS, Tokyo, Japan).

### 2.6. Western Blot Analysis

The total cell proteins were extracted using a Total Cell Protein Extraction Kit (Kaiji, Nanjing, China) according to the to the manufacturer's instructions. Protein (70 *μ*g) from each sample was loaded and separated by SDS-PAGE using 5% spacer gels and 10% separating gels. Proteins were transferred onto nitrocellulose membranes and then incubated overnight with the primary antibodies against iNOS, MR, Arg-1, VDR, PPAR*γ*, and *β*-actin at 4°C. After three washes with PBST/5 min, the nitrocellulose membranes were incubated with horseradish peroxidase-conjugated secondary antibody at a 1 : 5000 dilution for 1-2 h. Finally, the membranes were visualized with an enhanced chemiluminescence advanced system (GE Healthcare, UK) and captured on X-ray film. Immunoreactive bands were quantified with densitometry using Image J software (NIH, USA).

### 2.7. Statistical Analysis

All experiments were repeated at least three times. The data were expressed as the mean and standard deviation (SD) and were analyzed with SPSS 16.0. The differences of iNOS, MR, Arg-1, VDR, and PPAR*γ* among different groups were analyzed by one-way ANOVA. A difference was considered significant if the *P* value was less than 0.05.

## 3. Results

### 3.1. High Glucose Induces Macrophages toward an M1 Phenotype

**Figure 1:**
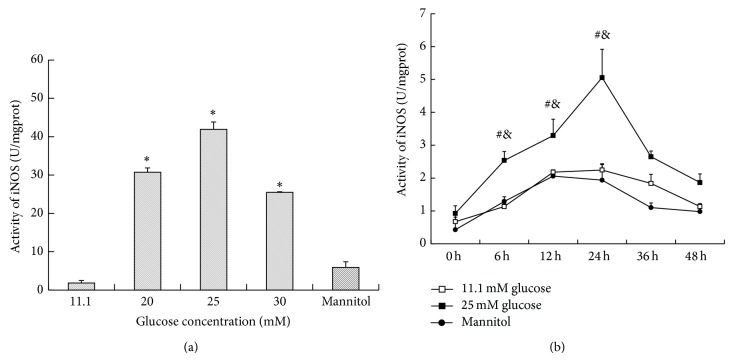
The effect of high glucose on the activity of iNOS. (a) RAW264.7 cells were stimulated with glucose in a dose- (11.1 mM, 20 mM, 25 mM, and 30 mM) dependent manner. After 24 h, the cells were collected. A concentration of 11.1 mM glucose was used as the control. ^*^ *P* < 0.05 versus control; (b) RAW264.7 cells were stimulated with glucose in a time- (0 h, 6 h, 12 h, 24 h, 36 h, and 48 h) dependent manner. A concentration of 11.1 mM glucose was used as the control. Data are presented as the mean ± SD (*n* = 3-4 per group). ^#^ *P* < 0.05 versus control at the same time point; ^&^ *P* < 0.05 versus 0 h in the same group.

**Figure 2:**
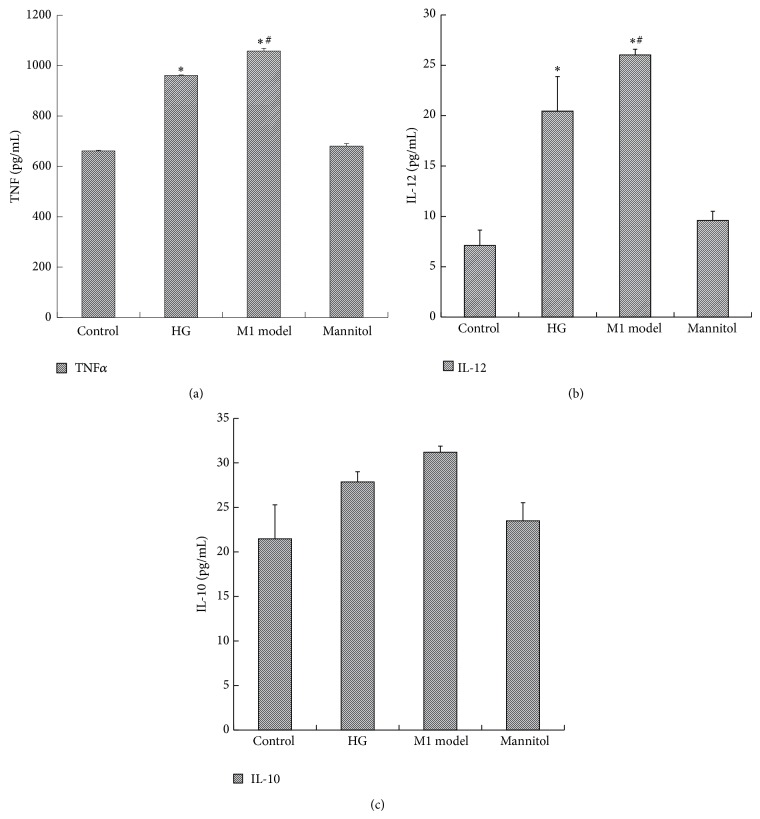
The effect of high glucose on the expression of cytokines in the supernatant. RAW264.7 cells were treated with 25 mM glucose (HG) for 24 h. The supernatant was collected for ELISA assay. A concentration of 11.1 mM glucose was used as the control. The M1 model group (100 U/mL IFN*γ* + 5 ng/mL LPS) was used as a positive control. Data are presented as the mean ± SD (*n* = 3-4 per group). ^*^ *P* < 0.05 versus control; ^#^ *P* < 0.05 versus HG.

**Figure 3:**
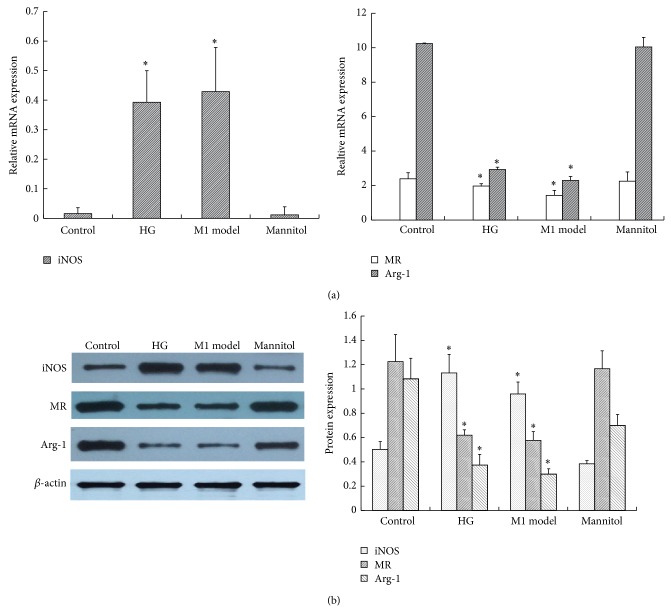
The effect of high glucose on M1/M2 macrophage-specific markers. RAW264.7 cells were treated with 25 mM glucose (HG) for 24 h. The cells were collected for (a) RT-PCR and (b) western blotting analysis. *β*-actin was used as an internal control. A concentration of 11.1 mM glucose was used as the control. The M1 model group (100 U/mL IFN*γ* + 5 ng/mL LPS) was used as a positive control. Data are presented as the mean ± SD (*n* = 3-4 per group). ^*^ *P* < 0.05 versus control.

In order to ascertain the optimum glucose concentration and time point, RAW264.7 cells were first stimulated with glucose in dose- (11.1 mM, 20 mM, 25 mM, and 30 mM) and time- (0 h, 6 h, 12 h, 24 h, 36 h, and 48 h) dependent manners, and the activity of inducible nitric oxide synthase (iNOS) was measured. As shown in Figure 1(a), the iNOS activity was increased by glucose in a dose-dependent manner. Particularly, 25 mM glucose gave the maximum response, and there was no difference between the control group and mannitol group, which excluded the effect of hyperosmolarity. As shown in Figure 1(b), from 0 h to 24 h, the iNOS activity increased in a time-dependent manner, and the peak level was achieved at 24 h after 25 mM glucose intervention. Then, there was a sharp decline in iNOS activity after 24 h. From 36 to 48 h, no significant difference in iNOS activity was found between high glucose and the control group. Thus, we used the 25 mM glucose concentration and 24 h time period in later experiments. Then, we explored the effect of glucose on macrophage phenotype by quantifying TNF-*α* and IL-12 in the supernatant and the expression of cell-specific markers of M1 and M2. As we can see, 25 mM glucose induced more secretion of inflammatory cytokines, including TNF-*α* and IL-12, in the supernatant, while anti-inflammatory IL-10 was not influenced (Figure 2 and Table 1). Similarly, when compared with the control, high glucose also stimulated high expression of an M1 marker, iNOS, but downregulated the expression of the M2 markers MR and Arg-1, which accorded with the classical activation model of M1 macrophages (Figure 3 and Table 2).

### 3.2. 1,25(OH)_2_D_3_ Polarizes High Glucose-Induced M1 Macrophages toward an M2 Phenotype

**Figure 4:**
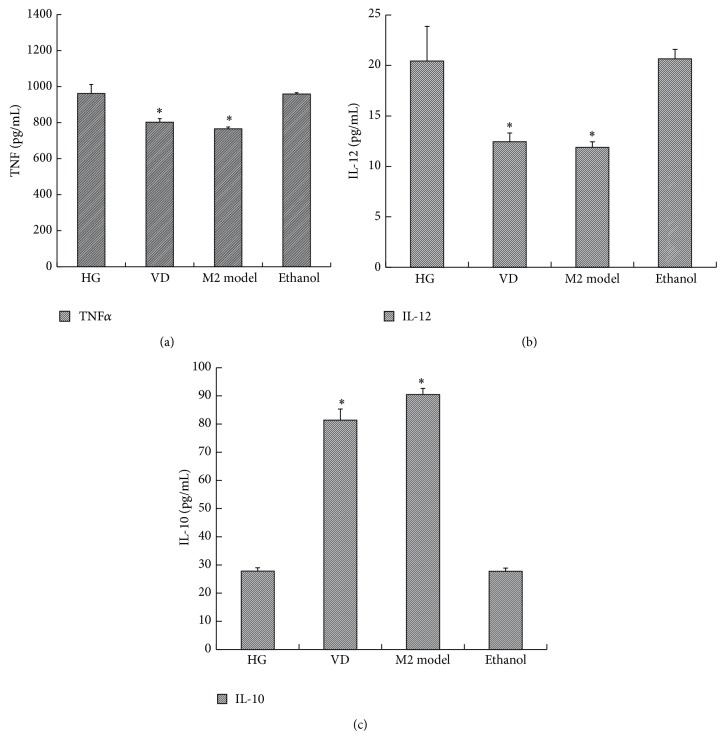
The effect of 1,25(OH)_2_D_3_ on the expression of cytokines in the supernatant. RAW264.7 cells were treated with 25 mM glucose (HG) in the presence or absence of 10^−8^ mol/L 1,25(OH)_2_D_3_ for 24 h. The supernatant was collected for ELISA assay. The M2 model group (10 ng/mL IL-4) was used as a positive control. Data are presented as the mean ± SD (*n* = 3-4 per group). ^*^ *P* < 0.05 versus HG.

**Figure 5:**
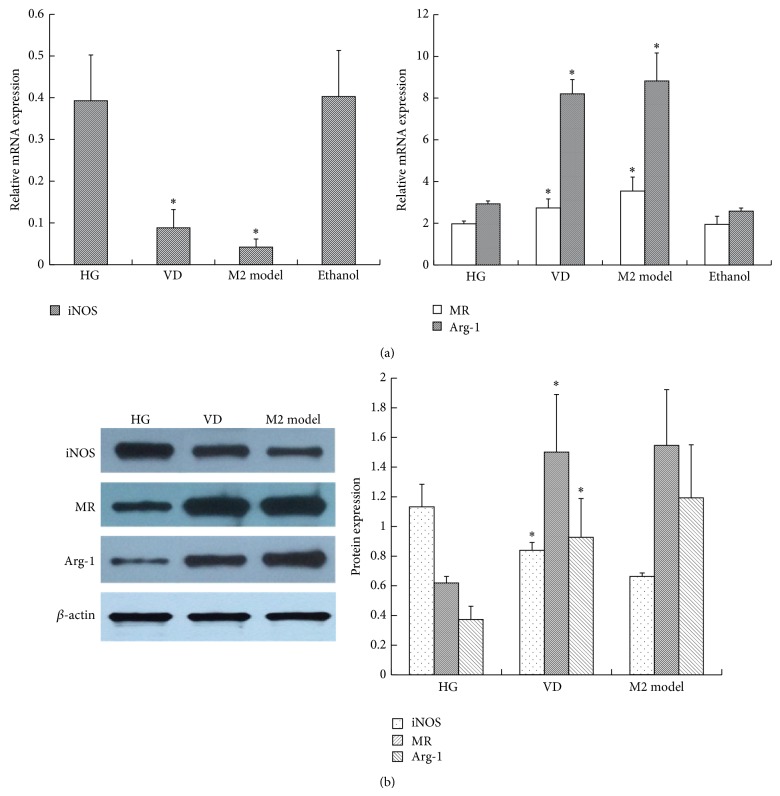
The effect of 1,25(OH)_2_D_3_ on M1/M2 macrophage-specific markers. RAW264.7 cells were treated with 25 mM glucose (HG) in the presence or absence of 10^−8^ mol/L 1,25(OH)_2_D_3_ for 24 h. The cells were collected for (a) RT-PCR and (b) western blotting analysis. *β*-actin was used as an internal control. The M2 model group (10 ng/mL IL-4) was used as a positive control. Data are presented as the mean ± SD (*n* = 3-4 per group). ^*^ *P* < 0.05 versus HG.

After 1,25(OH)_2_D_3_ exposure, TNF-*α* and IL-12 in the supernatant were evidently reduced, while IL-10 was increased when compared with the high glucose group. In addition, their expression was parallel to that of IL-4-induced M2 macrophage activation (Figure 4 and Table 1). Similar to these cytokines, high glucose-induced overexpression of iNOS was downregulated, yet the M2 markers MR and Arg-1 were significantly upregulated after 1,25(OH)_2_D_3_ stimulation (Figure 5 and Table 2). Consequently, the effect of high glucose-induced M1 polarization was blocked by 1,25(OH)_2_D_3_. Moreover, vitamin D further promoted them to an M2 phenotype.

### 3.3. 1,25(OH)_2_D_3_ Induces the Change from the M1 to M2 Macrophage Phenotype through the VDR-PPAR*γ* Pathway

#### 3.3.1. 1,25(OH)_2_D_3_ Promotes VDR and PPAR*γ* Expression

**Figure 6:**
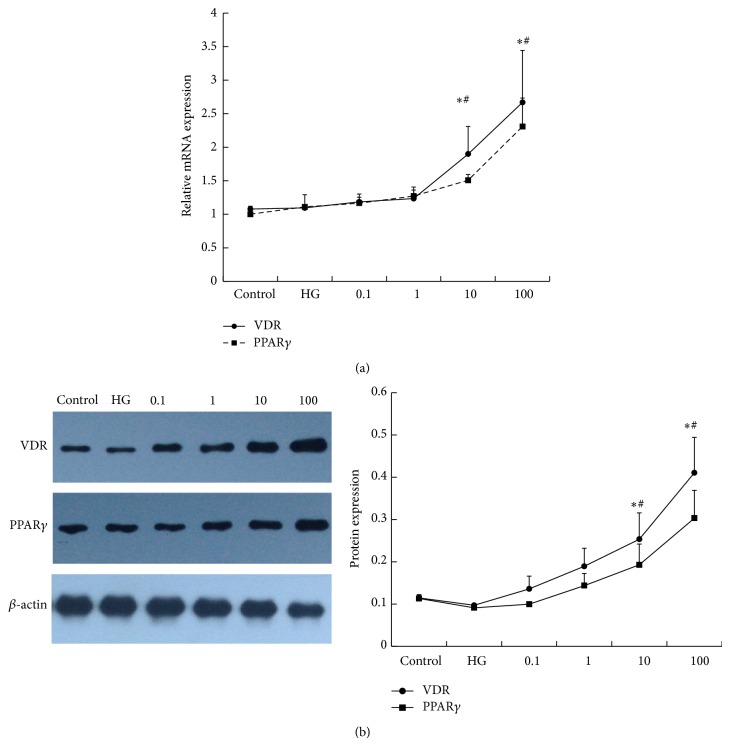
The effect of 1,25(OH)_2_D_3_ on the expression of VDR and PPAR*γ*. RAW264.7 cells were stimulated with 1,25(OH)_2_D_3_ in a dose- (0.1 nM, 1 nM, 10 nM, and 100 nM) dependent manner. After 24 h, the cells were collected for (a) RT-PCR and (b) western blotting analysis. *β*-actin was used as an internal control. A concentration of 11.1 mM glucose was used as a control. HG: 25 mM glucose. Data are presented as the mean ± SD (*n* = 3-4 per group). ^*^ *P* < 0.05 versus control; ^#^ *P* < 0.05 versus HG.

The biological effects of 1,25(OH)_2_D_3_ are mediated through a nuclear hormone receptor known as the vitamin D receptor (VDR), and PPAR*γ* has been suggested to partake in regulating macrophage phenotype. Thus, we explored the expression and interaction of the two receptors. In our study, we found that VDR mRNA was increased by vitamin D in a dose-dependent manner. However, the extent was not significant with 10^−10^ mol/L (1.18 ± 0.12) and 10^−9^ mol/L (1.23 ± 0.17) vitamin D stimulation when compared with the control (1.07 ± 0.04). In contrast, 10^−8^ mol/L (1.90 ± 0.41) and 10^−7^ mol/L (2.67 ± 0.78) vitamin D obviously upregulated the expression of VDR mRNA, not only with the control but also with the high glucose-treated cells. The VDR protein level showed the same trend. Additionally, PPAR*γ* caused an identical effect as VDR (Figure 6).

#### 3.3.2. PPAR*γ* Antagonist Abolished the Effect of 1,25(OH)_2_D_3_

**Figure 7:**
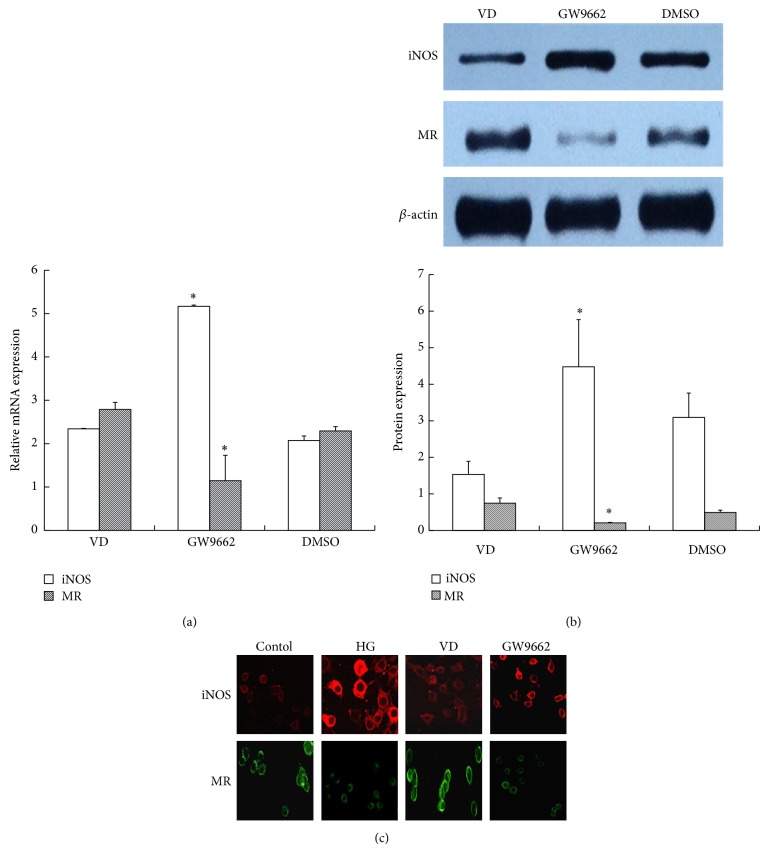
The effect of a PPAR*γ* antagonist (GW9662) on iNOS and MR expression. RAW264.7 cells were treated with GW9662 for 2 h before administering 10^−8^ M 1,25(OH)_2_D_3_ to high glucose-pretreated macrophages. The cells were collected for (a) RT-PCR, (b) western blotting, and (c) immunofluorescence analysis (200x). *β*-actin was used as an internal control. DMSO was used as a negative control. Data are presented as the mean ± SD (*n* = 3-4 per group). ^*^ *P* < 0.05 versus VD group.

To further determine the role of PPAR*γ* in the regulation of macrophage phenotype, a PPAR*γ* antagonist (GW9662) was used to stimulate the vitamin D-pretreated, high glucose-incubated cells. As showed in Figure 7(a), after pretreatment with GW9662 for 2 h, the expression of MR mRNA, which was enhanced by vitamin D, was decreased (VD versus GW9662: 2.79 ± 0.16 versus 1.14 ± 0.09), while iNOS mRNA was increased when compared with the vitamin D group (VD versus GW9662: 2.34 ± 0.01 versus 5.17 ± 0.03). The protein level indicated the same change (Figure 7(b)). Immunofluorescence staining also showed enhanced iNOS but weak MR fluorescent expression in the GW9662 group (Figure 7(c)). The above results suggested that the vitamin D-induced macrophage M2 polarization was abolished.

#### 3.3.3. VDR siRNA Blocked the Effect of 1,25(OH)_2_D_3_

**Figure 8:**
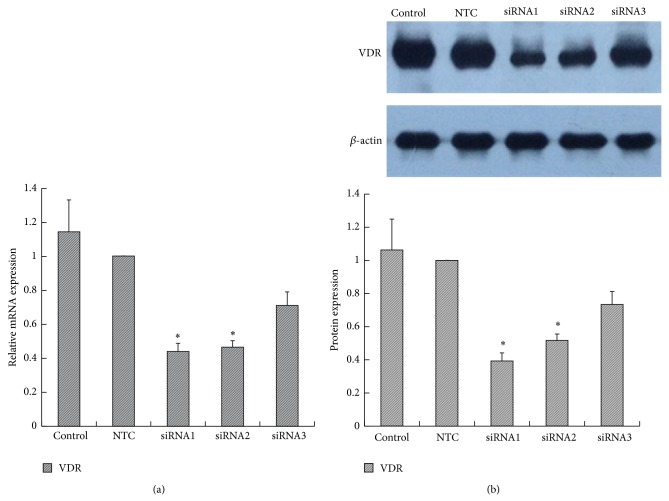
The effect of VDR siRNA on VDR expression. RAW264.7 cells were transfected with three specific VDR siRNAs or a nontarget control (NTC) siRNA. The VDR gene (a) and protein (b) levels were measured. *β*-actin was used as an internal control. Data are presented as the mean ± SD (*n* = 3-4 per group). ^*^ *P* < 0.05 versus control.

**Figure 9:**
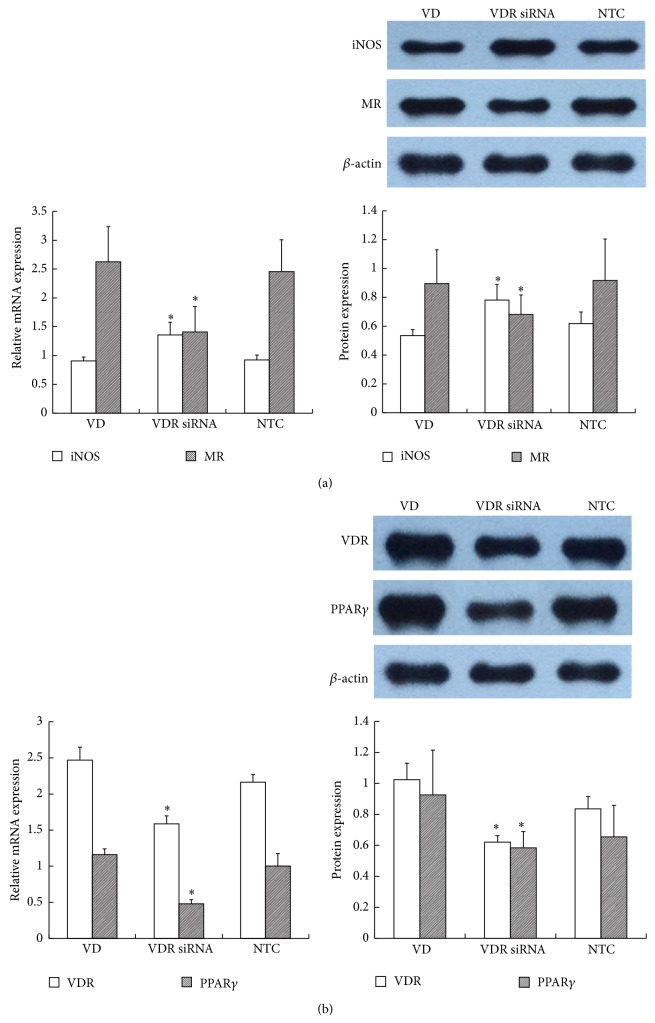
The effect of VDR siRNA on iNOS, MR, and PPAR*γ* expression. VDR siRNA pretreatment with cells was performed before administering 10^−8^ M 1,25(OH)_2_D_3_ to high glucose-treated macrophages. The cells were collected for (a) iNOS, MR, and (b) PPAR*γ* analysis. *β*-actin was used as an internal control. NTC: nontarget control. Data are presented as the mean ± SD (*n* = 3-4 per group). ^*^ *P* < 0.05 versus VD group.

**Figure 10:**
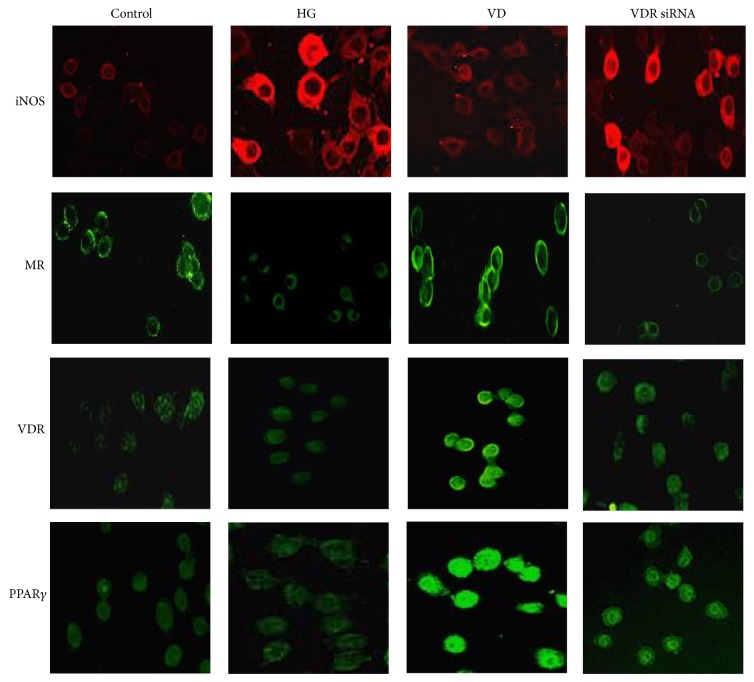
The iNOS, MR, VDR, and PPAR*γ* immunofluorescence expression after VDR siRNA intervention (200x).

siRNA targeting VDR was transfected into RAW264.7 cells. A non-target control (NTC) siRNA was used to eliminate the nonspecific effects of the transfection reagents. All three specific VDR siRNAs inhibited VDR expression, but significant differences appeared only with VDR siRNA-1 (0.44 ± 0.05) and VDR siRNA-2 (0.47 ± 0.04) when compared with the control (1.15 ± 0.19) and NTC (1.00 ± 0.00) groups. The inhibition ratios of VDR siRNA-1, 2, and 3 were 56%, 53.5%, and 29.0%, respectively, so we used VDR siRNA-1 as the final intervention siRNA. NTC siRNA clearly showed no effect on VDR expression (Figure 8). As shown in Figure 9(a), depletion of VDR blocked the 1,25(OH)_2_D_3_-mediated increase in MR mRNA (VD versus VDR siRNA: 2.63 ± 0.61 versus 1.41 ± 0.44) and decrease in iNOS mRNA expression (VD versus VDR siRNA: 0.91 ± 0.07 versus 1.36 ± 0.22), indicating that suppression of VDR expression eliminated the 1,25(OH)_2_D_3_-induced M1 macrophage switch to M2. The protein level showed the same change as the gene level. In addition, we further found that PPAR*γ* was synchronously decreased after inhibition of VDR expression, which indicated that PPAR*γ* may locate downstream of the vitamin D signaling pathway (Figure 9(b)). Immunofluorescence staining showed the change of each marker (Figure 10). In conclusion, the above results suggested that VDR-PPAR*γ* cross talk may play an important role in the regulation of macrophage activation by 1,25-dihydroxyvitamin D_3_.

## 4. Discussion

Diabetic nephropathy (DN) is the leading cause of end-stage renal disease [27]. Many studies have explored the pathogenesis of DN, hyperglycemia, advanced glycation end products (AGEs), and oxidative stress, all of which, to some extent, participate in the occurrence and development of DN [28]. However, the exact biochemical and molecular mechanism is complex and is still not fully elucidated. Inflammation has been recently identified in the evolvement of DN, and macrophages show a central role in the process. Chow et al. found that progressive DN injury in db/db mice was associated with an increase in kidney macrophages. Macrophage accumulation and activation in db/db mice were correlated with albuminuria, glomerular and tubular damage, renal fibrosis, and proinflammatory chemokines [29]. To extend beyond animal models, Nguyen also suggested a pathogenic role of macrophages in human DN, and the glomerular and interstitial macrophage number was correlated strongly with serum creatinine level, proteinuria, and interstitial fibrosis and was proportional to the rate of subsequent decline in renal function [30]. Our previous study also found that streptozocin- (STZ-) induced DN rats showed more infiltrating macrophages, thickened glomerular mesangial matrix, aggravated podocyte injury, proteinuria, and progressive decline of renal function [16, 31]. Consequently, we long considered the quantity of infiltrating macrophages to reflect the development of DN. However, as the investigation of macrophage phenotype and function has progressed, the conventional viewpoint is being challenged.

Macrophages comprise a heterogeneous population of cells that belong to the mononuclear phagocyte system. During enhanced recruitment in response to disease states, inflammatory monocytes are recruited in response to cytokine cues and undergo differentiation into two broad but distinct subsets of macrophages that are categorized as either classically activated (M1) or alternatively activated (M2). The two different subsets demonstrate antigenic and functional heterogeneity [2]. Exposure to IFN-*γ* and LPS or GM-CSF induces M1 polarization that is characterized by the production of inducible nitric oxide synthase (iNOS), tumor necrosis factor *α* (TNF*α*), interleukin-1 (IL-1), IL-12, and reactive oxygen species (ROS). All of these contribute to inflammation and exacerbate renal injury. M2 macrophages represent various phenotypes that are further subdivided into M2a (upon exposure to IL-4 or IL-13), M2b (induced by immune complexes in combination with IL-1*β* or LPS), and M2c cells (following exposure to IL-10, TGF-*β*, or glucocorticoids). They are thought to suppress immune responses, inhibit inflammation, and promote tissue remodeling [32]. Mannose receptor (MR) and arginase-1 (Arg-1) are representative markers. Lee et al. suggested that, in the first 48 hours after ischemia/reperfusion injury, it was M1 macrophages that recruited into the kidney, which can promote inflammation and worse renal injury, while, during the period of tubular cell proliferation and recovery, noninflammatory (M2) macrophages predominated [11]. Wang et al. indicated that adoptive transfer of macrophage primed ex vivo by exposure to IL-4 and IL-13 to induce M2 macrophage can reduce renal injury and facilitate repair in adriamycin nephropathy mice [33]. Our previous study also found that, in streptozocin- (STZ-) induced DN rats, there was an increased number of M1 macrophages that infiltrated in the glomeruli and interstitium, followed by aggravated renal histopathologic changes, podocyte loss, increased proteinuria, and deterioration of renal function, while M2 macrophages inhibited inflammation and alleviated podocyte impairment and proteinuria, finally promoting renal recovery [16]. All of the above results revealed it was the macrophage activation state, but not numbers, that finally indicated the development and prognosis of DN. This was further confirmed by the present in vitro study, in which, under high glucose conditions, RAW264.7 cells exhibited an M1 phenotype, expressing high iNOS and the proinflammatory cytokines TNF*α* and IL-12 but with inhibition of M2 markers.

Numerous studies have explored methods to switch macrophage phenotype, including genetic modification and ex vivo venous transfusion, but these current strategies are not realistic clinically [32]. As a result, finding more practical ways to regulate macrophage phenotype is of great concern. 1,25(OH)_2_D_3_ is an endocrine hormone with multiple physiological functions. The activity of 1,25(OH)_2_D_3_ is mediated by VDR [34]. 1,25(OH)_2_D_3_-VDR has multiple physiological and pathological roles that extend beyond the regulation of mineral metabolism, including the regulation of renal and cardiovascular functions [35]. Numerous studies have proven the renoprotective role of vitamin D in various kidney diseases through preventing podocyte dysfunction, alleviating albuminuria, and ameliorating renal fibrosis [36]. Our study also indicated that calcitriol, a bioactive 1,25-dihydroxyvitamin D_3_, could markedly inhibit podocyte foot process effacement and decrease the glomerular basement thickness, resulting in attenuating albuminuria and preventing the decline of renal function in diabetic nephropathy [15, 31]. Furthermore, this protective role may relate to the regulation of macrophage phenotype. In the early stage of DN, vitamin D inhibits M1 macrophage infiltration, and, later, it promotes M2 macrophage activation [16]. The present in vitro study confirmed such changes as well. After vitamin D treatment, proinflammatory cytokines in the supernatant, including TNF*α*, IL-12, and the M1 marker iNOS, were decreased, while M2 markers, MR and Arg-1, were increased, suggesting that vitamin D could switch high glucose-induced M1 macrophages toward an M2 phenotype. Besides, we found that vitamin D alone had no such effect on macrophage phenotype; this was also in accord with our in vivo experiment (data not shown).

It has been shown that the activity of 1,25(OH)_2_D_3_ is mediated by VDR, and our in vivo study found that the renoprotective effect of vitamin D in DN rats could be attributed to the enhancement of VDR [31]. In the present study, VDR was upregulated after treatment with vitamin D. Additionally, we found that another ligand-activated nuclear receptor transcriptional factor, PPAR*γ*, was also increased. When either of them was inhibited, the effect of vitamin D on regulating macrophage phenotype was also blocked. In addition, the effect of PPAR*γ* was further abolished when VDR expression was blocked. This suggested that the VDR-PPAR*γ* pathway may be the underlying mechanism behind the regulation of macrophage phenotype by vitamin D. This coincided with other findings that macrophage-specific PPAR*γ* was a specific factor that controls M2 macrophage activation [20]; in addition, VDR and PPAR*γ* can interact with each other in a variety of other cells [23, 24].

## 5. Conclusions

The present study demonstrates that 1,25-dihydroxyvitamin D_3_ can switch high glucose-induced M1 macrophages to M2 ex vivo, and, for the first time, we present evidence that the VDR-PPAR*γ* pathway may play a decisive role during this conversion. Altogether, these findings contribute to the understanding of the renoprotective effect of 1,25-dihydroxyvitamin D_3_ in diabetic nephropathy.

## Figures and Tables

**Table 1:** The expression of cytokines in the supernatant of each group (ELISA, pg/mL).

	Control	HG	M1 model	Mannitol	VD	M2 model
TNF-*α*	660.84 ± 3.21	960.82 ± 1.99^*^	1057.64 ± 11.42^*^	680.14 ± 10.38	802.00 ± 11.37^#^	765.20 ± 33.76^#^
IL-12	7.11 ± 1.54	20.45 ± 3.43^*^	26.04 ± 0.56^*^	9.60 ± 0.91	12.45 ± 0.86^#^	11.87 ± 0.56^#^
IL-10	21.47 ± 3.83	27.85 ± 1.14	31.19 ± 0.68	23.48 ± 2.03	81.40 ± 3.91^#^	90.47 ± 2.22^#^

Control: 11.1 mM glucose; HG: 25 mM glucose; M1 model: 100 U/mL IFN*γ* + 5 ng/mL LPS; VD: 25 mM glucose + 10^−8^ mol/L 1,25(OH)_2_D_3_; M2 model: 10 ng/mL IL-4. Data are presented as the mean ± SD (*n* = 3-4 per group). ^*^ *P* < 0.05 versus control; ^#^ *P* < 0.05 versus HG.

**Table 2:** The mRNA expression of M1/M2 macrophage-specific markers of each group (RT-PCR).

	Control	HG	M1 model	Mannitol	VD	M2 model
iNOS	0.02 ± 0.005	0.39 ± 0.11^*^	0.43 ± 0.15^*^	0.01 ± 0.003	0.09 ± 0.04^#^	0.04 ± 0.02^#^
MR	2.39 ± 0.36	1.97 ± 0.13^*^	1.43 ± 0.30^*^	2.26 ± 0.54	2.74 ± 0.03^#^	3.54 ± 0.12^#^
Arg-1	10.24 ± 0.03	2.93 ± 0.14^*^	2.29 ± 0.24^*^	10.04 ± 0.56	8.20 ± 0.68^#^	8.82 ± 1.34^#^

Control: 11.1 mM glucose; HG: 25 mM glucose; M1 model: 100 U/mL IFN*γ* + 5 ng/mL LPS; VD: 25 mM glucose + 10^−8^ mol/L 1,25(OH)_2_D_3_; M2 model: 10 ng/mL IL-4. Data are presented as the mean ± SD (*n* = 3-4 per group). ^*^ *P* < 0.05 versus control; ^#^ *P* < 0.05 versus HG.
